# Association of early-onset Alzheimer’s disease with germline-generated high affinity self-antigen load

**DOI:** 10.1038/s41398-020-0826-6

**Published:** 2020-05-12

**Authors:** Poyin Huang, Yuan-Han Yang, Ya-Hsuan Chang, Shu-Ling Chang, Mei-Chuan Chou, Chiou-Lian Lai, Ching-Kuan Liu, Hsuan-Yu Chen

**Affiliations:** 1 Department of Neurology, Kaohsiung Medical University Hospital, Kaohsiung Medical University, Kaohsiung, Taiwan; 2 Department of Neurology, Kaohsiung Municipal Siaogang Hospital, Kaohsiung Medical University, Kaohsiung, Taiwan; 3 Neuroscience Research Center, Kaohsiung Medical University, Kaohsiung, Taiwan; 4 Department of Neurology, Faculty of Medicine, College of Medicine, Kaohsiung Medical University, Kaohsiung, Taiwan; 5 Department of Neurology, Kaohsiung Municipal Ta-Tung Hospital, Kaohsiung, Taiwan; 6 Institute of Statistical Science, Academia Sinica, Taipei, Taiwan; 7 School of Post-Baccalaureate Medicine, Kaohsiung Medical University, Kaohsiung, Taiwan; 8 Ph.D. Program in Microbial Genomics, National Chung Hsing University, Taichung, Taiwan

**Keywords:** Clinical genetics, Clinical genetics, Molecular neuroscience

## Abstract

Self-antigen presentation outside the central nervous system has crucial role regarding self-proteins tolerance and autoimmunity, leading to neuroinflammation. Self-antigen with strong-binding affinity is considered to be pathogenic. We aim to investigate whether strong-binding affinity self-antigen load is associated with early/late-onset Alzheimer’s disease (AD). A total of 54 AD samples (22 early-onset, 32 late-onset) underwent next-generation sequencing (NGS) for whole-exome sequencing. Genotypes of HLA class I genes and germline mutations were obtained for estimation of the binding affinity and number of self-antigens. For each patient, self-antigen load was estimated by adding up the number of self-antigens with strong-binding affinity. Self-antigen load of early-onset AD was significantly higher than late-onset AD (mean ± SD: 6115 ± 2430 vs 4373 ± 2492; *p* = 0.011). An appropriate cutoff value 2503 for dichotomizing self-antigen load was obtained by receiver operating characteristic (ROC) curve analysis. Patients were then dichotomized into high or low self-antigen load groups in the binary multivariate logistic regression analysis. Adjusted odds ratio of the high self-antigen load (>2503) was 14.22 (95% CI, 1.22–165.70; *p* = 0.034) after controlling other covariates including gender, education, ApoE status, and baseline CDR score. This is the first study using NGS to investigate germline mutations generated self-antigen load in AD. As strong-binding affinity self-antigen is considered to be pathogenic in neuroinflammation, our finding indicated that self-antigen load did have a role in the pathogenesis of AD owing to its association with neuroinflammation. This finding may also contribute to further research regarding disease mechanism and development of novel biomarkers or treatment.

## Introduction

Alzheimer’s disease (AD) is the most common cause of cognitive decline of the elderly. AD is pathologically defined by the accumulation of extracellular β-amyloid plaques and intracellular neurofibrillary tangles in the brain along with extensive neuronal loss^1^. However, increasing evidence also shows that AD pathogenesis is not only limited to β-amyloid plaques and intracellular neurofibrillary tangles, but also includes strong immunological interactions within the brain^2^.

The accumulation of β-amyloid plaques and toxic tau protein could activate microglia and induce innate immune responses characterized by release of inflammatory mediators, which further contributes to AD progression^3^.

In human genome, the human leukocyte antigen (HLA) gene contains the most polymorphic region, as it codes for antigens directly involved in immunological recognition. The high polymorphism of HLA gene is responsible for the complicated ability of the immune system to react to a tremendous variety of different peptides^4,5^. As immune responses are based on the capability of HLA molecules to differentially bind specific peptides, the strength of immune responses differs according to the presentation of variable HLA antigens. Thus, specific HLA molecules induce distinct immune responses and may have a role in regulating susceptibility to certain diseases such as AD, supported by some recent studies showing that some specific HLA alleles are involved in the pathogenesis of AD^6–9^. Furthermore, striking evidences indicate that the immune system is intrinsically involved in the pathogenesis of AD. Auto-antibodies against a variety of molecules have been identified in AD patients, but the roles of these auto-antibodies in the pathogenesis of AD are yet unclear. Many concepts regarding central nervous system (CNS) immune function have been challenged and changed in recent years, including but not limited to the discoveries of CNS-draining lymphatic vessels, the origin and functional diversities of microglia, the influence of T cells on CNS homeostasis to immunology, and the role neuroinflammation plays in neurodegenerative diseases^10^. On the other hand, self-antigen presentation outside the CNS may have crucial role regarding self-proteins tolerance and autoimmunity leading to neuroinflammation^11^. Based on the above theories, we hypothesize that germline mutation generated self-antigens will have a negative role in AD, to the opposite of somatic mutation generated antigens, which play a positive role in cancer treatment. The aim of this study is to utilize cutting edge next-generation sequencing (NGS) technique, the whole-exome sequencing, to generate HLA-based self-antigen load in AD patients and test whether it has a role in AD.

## Materials and methods

### Study population and DNA extraction

A total of 54 AD patients were collected from the neurological outpatient department of Kaohsiung Medical University Hospital. The diagnosis of AD was based on the National Institute of Neurological and Communicative Disorders and Stroke and Alzheimer’s Disease and Related Disorders Association (NINCDS-ADRDA) criteria. The patients with AD had to fulfill the following criteria in order to be included in the probable group in this study: (1) a diagnosis of dementia established by clinical and neuropsychological examinations; (2) progressive cognitive impairments present in two or more areas of cognition without conscious disturbance; (3) onset after the age of 40 and before the age of 90 years; and (4) brain CT or MRI studies compatible with AD. The included patients were taking donepezil 5–10 mg/d, rivastigmine 3–6 mg/12 hours, galantamine 8–12 mg/12 hours, or memantine 5–10 mg/12 hours. An annual interview was performed with each patient, and the Clinical Dementia Rating (CDR) score (0 (normal) to 3 (severe stage)) was used to evaluate their functional deterioration. The patients with an increased CDR score during follow-up were defined as having clinical progression. The clinical characteristics including age, gender, education level, ApoE genotype, serial CDR score, baseline stages of AD, and medications used for AD were recorded. Genomic DNA was extracted from the buffy coat following standard protocols.

This study was approved by the Institutional Review Board of our hospital, and written informed consent was obtained from all patients or their relatives before participating in this study. The above method has been described in detail in our previous publication^12^.

### Whole-exome sequencing analysis and HLA genotyping

The NGS platform HiSeq 4000 (Illumina, San Diego, CA) was performed for whole-exome sequencing analysis with SureSelect Human All Exon V6 kit (Agilent Technologies, Santa Clara, CA). The adaptor sequences were first removed from the raw sequencing reads data. In the quality control for all reads, lower-quality bases (sliding window with four-base length if average quality with the window <20 base quality) were further trimmed from the raw sequence reads using software Trimmomatic (v0.33). Trimmed reads were mapped to hg19 human reference genome using Burrows-Wheeler Aligner (v0.7.5a). In order to reduce false positive results, realignment, quality score recalculation and marking duplication possible from PCR were done by GATK (v3.7) and Picard (v1.98). After the quality controls, removed duplications, and, mapping results, the average reads throughput was 100× depth. The HaplotypeCaller method of GATK was used to detect germline mutation. Based on the germline mutations of each sample, the Polysolver software was carried out to estimate genotypes of HLA class I genes- A, B, and C, respectively. This algorithm extracted reads in HLA region and aligned them to the database of HLA full-length genomic sequences in the international immunogenetics information system (http://www.imgt.org). Based on the read alignment status, insert size distribution, base qualities, and information of Asian population allele frequency distribution, each sample’s class I HLA genotypes were estimated.

### HLA genotyping of Taiwanese general population

In order to obtain HLA-A, HLA-B, and HLA-C genotypes of Taiwanese general population for comparison with AD patients, genotypes of HLA class I genes were retrieved from Taiwan Biobank (https://www.twbiobank.org.tw/new_web_en/index.php).

A total of 1075 individuals with HLA genotype data were available for HLA-A, 1090 individuals were available for HLA-B and 1089 individuals were available for HLA-C. These HLA genotypes were then used to compare with HLA genotypes of AD patients.

### Prediction of the binding affinity of self-antigen

**Fig. 1:**
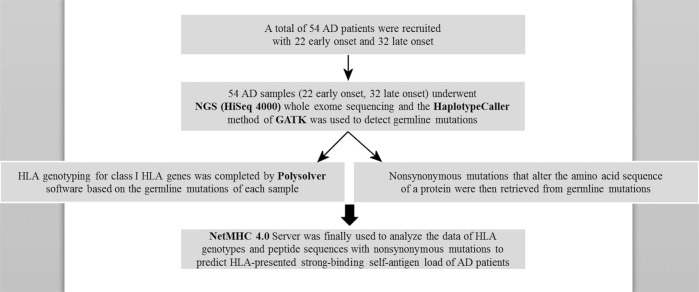
NGS analysis for prediction of self-antigen load. Flowchart of AD samples underwent whole-exome sequencing to acquire high affinity strong-binding self-antigen load. The details of the tools used were described in the methods section.

Based on the detected germline mutations, ANNOVAR software was used to annotate identified variants. After the above analysis, germline mutations that could cause peptide change (nonsynonymous mutations) were further retrieved. After the annotation step, each nonsynonymous mutation was annotated how amino-acid changing and the mutant position of the protein (e.g., YAP1 R331W was amino acid R to W changed in the position 331 on protein YAP1). Because the binding peptide length of HLA class 1 was nearly 8–14 amino acids, a total of 13 amino acids on upper and downstream of the mutant site were selected. Hence, the length of the selected peptide was 27 amino acids (e.g., for YAP1 R331W, selected peptide was from position 318–344 and centered on 331). Finally, nonsynonymous mutations derived peptide sequences and HLA genotypes were further input to NetMHC 4.0 Server (http://www.cbs.dtu.dk/services/NetMHC/) to predict binding affinity of self-antigen for AD patients that could be presented by HLA (Fig. 1). The number of the peptides with strong-binding affinity was the self-antigen load and the unit of the self-antigen load was peptide number.

### Statistical analysis

The Fisher’s exact test or Mann–Whitney *U* test was used to compare differences between groups for categorical or continuous variables, respectively. Receiver operating characteristic (ROC) curve was used to find an appropriate cutoff value for self-antigen load. Binary logistic regression was applied for multivariate analysis using early/late-onset as dependent variable while self-antigen load, gender, years of education, baseline CDR score, and ApoE genotype were added to the model as covariates. For the power calculation, given the sample sizes for early and late-onset AD = 22 and 32, type 1 error = 0.02, and crude odds ratio = 12.6, the power of this study is 0.78. It is close to 0.8, which is the commonly used power value. All tests were two-sided and a *p* value < 0.05 was considered significant.

## Results

**Fig. 2:**
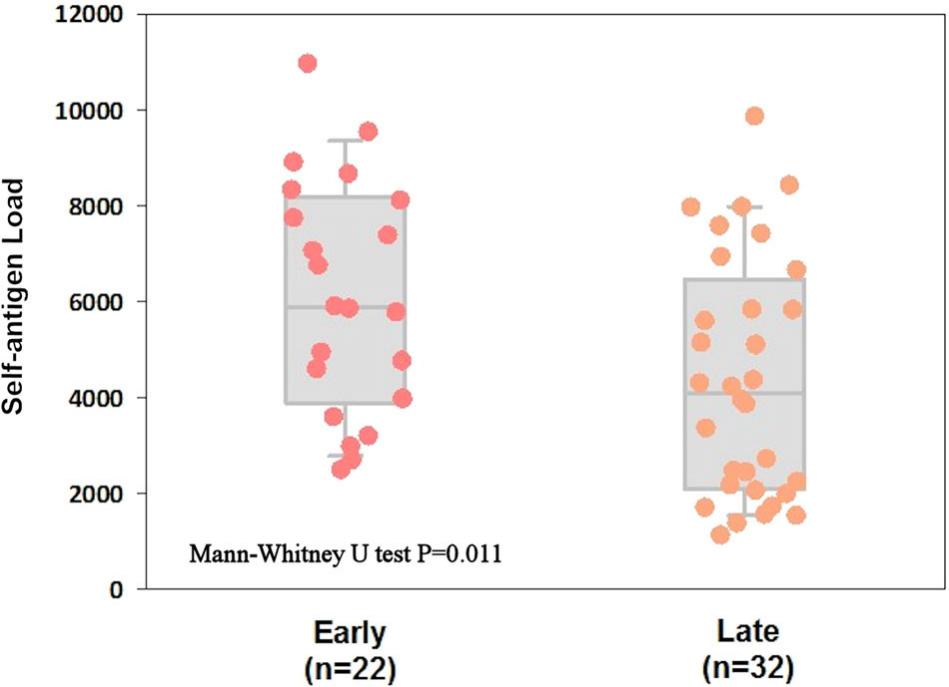
Self-antigen load distribution. Distributions of high affinity strong-binding self-antigen load of early-onset AD versus late-onset AD. The differences were compared using Mann–Whitney *U* test.

A total of 22 early-onset and 32 late-onset AD patients were recruited in this study for analysis. All patients were Han Chinese and no relationship between samples. The basic characteristics of these 54 patients were shown in Table 1. In the early-onset group, average education years were borderline higher (8.88 ± 3.83 vs 6.38 ± 4.95; *p* = 0.084) and baseline dementia stage of the diagnosis time was more severe (lower percentage of CDR 0.5–1, 68.2% vs 96.9%; *p* = 0.006). In addition, early-onset group had more patients with high self-antigen load (95.45%) than late-onset group (62.5%) (*p* = 0.008). There were no significant differences between gender and the status of ApoE ε4+. However, self-antigen load of early-onset AD was significantly higher than that of late-onset (average ± SD: 6115 ± 2430 vs 4373 ± 2492; *p* = 0.011) (Table 1 and Fig. 2).

**Table 1:** Basic characteristics of AD patients (*n* = 54) underwent next-generation sequencing analysis.

	Early onset (*n* = 22)	Late onset (*n* = 32)	*P* value
Age (year)	60.86 ± 3.22	76.28 ± 5.77	<0.001
Gender (male)	8 (36.4%)	9 (28.1%)	0.563
Education (year)	8.88 ± 3.83	6.38 ± 4.95	0.084
Self-antigen load	6115 ± 2430	4373 ± 2492	0.011
High self-antigen load	21 (95.5%)	20 (62.5%)	0.008
ApoE (ε4 + )	7 (31.8%)	16 (50.0%)	0.264
Baseline CDR (0.5 or 1)	15 (68.2%)	31 (96.9%)	0.006

We also obtained HLA class I genotypes of Taiwanese general population (1075 cases for HLA-A, 1090 cases for HLA-B, and 1089 cases for HLA-C) from Taiwan Biobank. Alleles of HLA-A, HLA-B, and HLA-C were compared between AD patients and general population. Results showed that HLA-A*11:01 (28.7% and 25.2%), HLA-B*40:01 (21.3% and 18.8%) and HLA-C*07:02 (33.3% and 19.4%) (Table 2) were the most major HLA class I genotypes for both AD patients and general population. When using HLA genotype as a binominal variate (for example, HLA-A*02:01 vs non HLA-A*02:01) for further analysis between early and late-onset AD (categorical, early vs late), we found that HLA-A*02:01 (early-onset 31.8% vs late-onset 6.3%; *p* = 0.023) was identified to be associated with the age of AD onset.

**Table 2:** Comparison of HLA class I allele frequencies between AD cases and general population (GP).

HLA-A allele	AD (*n*/%) allele number = 108	GP (*n*/%) allele number = 2150	*P* value
*02:01	11/10.2%	224/10.4%	0.001
*02:03	11/10.2%	131/6.1%	
*02:07	14/13.0%	185/8.6%	
*11:01	31/28.7%	542/25.2%	
*11:02	6/5.5%	86/4.0%	
*24:02	16/14.8%	368/17.1%	
*26:01	2/1.8%	63/2.9%	
*29:01	1/0.9%	3/0.1%	
*30:01	3/2.8%	48/2.2%	
*31:01	1/0.9%	58/2.7%	
*32:01	1/0.9%	15/0.7%	
*33:03	9/8.3%	279/13%	
*34:01	1/0.9%	1/0.0%	
*68:01	1/0.9%	3/0.1%	
*02:06	0	67/3.1%	

HLA-B allele	AD (*n*/%) allele number = 108	GP (*n*/%) allele number = 2180	*P* value
*07:05	2/1.8%	1/0.0%	0.001
*08:01	2/1.8%	5/0.2%	
*13:01	6/5.5%	122/5.6%	
*13:02	2/1.8%	51/2.3%	
*15:01	4/3.7%	83/3.8%	
*15:02	1/0.9%	80/3.7%	
*15:07	1/0.9%	1/0.0%	
*15:11	1/0.9%	23/1.1%	
*15:18	1/0.9%	14/0.6%	
*15:25	1/0.9%	18/0.8%	
*27:04	1/0.9%	57/2.6%	
*35:01	6/5.5%	66/3.0%	
*38:02	11/10.2%	97/4.4%	
*39:01	3/2.8%	47/2.2%	
*40:01	23/21.3%	410/18.8%	
*40:02	5/4.6%	57/2.6%	
*40:06	2/1.8%	38/1.7%	
*44:02	1/0.9%	10/0.5%	
*46:01	11/10.2%	235/10.8%	
*48:01	3/2.8%	22/1.0%	
*51:01	2/1.8%	97/4.4%	
*52:01	1/0.9%	30/1.4%	
*54:01	2/1.8%	72/3.3%	
*55:02	2/1.8%	58/2.7%	
*56:03	2/1.8%	7/0.3%	
*58:01	11/10.2%	262/12%	
*67:01	1/0.9%	8/0.4%	

HLA-C allele	AD (*n*/%) allele number = 108	GP (*n*/%) allele number = 2178	*P* value
*01:02	20/18.5%	354/16.3%	0.001
*03:02	12/11.1%	263/12.1%	
*03:03	10/9.3%	126 /5.8%	
*03:04	11/10.2%	242/11.1%	
*04:01	1/0.9%	93/4.3%	
*04:03	2/1.8%	38/1.7%	
*05:01	1/0.9%	8/0.4%	
*06:02	2/1.8%	72/3.3%	
*07:02	36 /33.3%	423/19.4	
*08:01	4/3.7%	150 /6.9%	
*12:02	3/2.8%	88/4.0%	
*14:02	2/1.8%	79/3.6%	
*15:02	3/2.8%	107/4.9%	
*15:05	1/0.9%	1/0.0%	

ROC curve was further performed to find that self-antigen load 2503 was an appropriate cutoff value for binary multivariate logistic regression analysis (Supplementary Fig. 1). Patients were dichotomized into high or low self-antigen load groups in the binary multivariate logistic regression analysis (Table 3). Adjusted odds ratio of the high self-antigen load (>2503) was 14.22 (95% CI, 1.22–165.70; *p* = 0.034) after controlling other covariates including gender, education, ApoE status, and baseline CDR score.

**Table 3:** Adjusted odds ratios for the early-onset AD.

Variable	Odds ratio (95% CI)	*P* value
Gender (male)	0.46 (0.08–2.62)	0.384
Education (year)	1.20 (0.99–1.46)	0.065
High self-antigen load^a^	14.22 (1.22–165.70)	0.034
ApoE (ε4+)	0.31 (0.07–1.40)	0.126
Baseline CDR (0.5 or 1)	0.04 (0.002–0.68)	0.026

^a^The cutoff value was 2503 estimated from receiver operating characteristic curve analysis, *CI* confidence interval.

## Discussion

In the past one to two decades of AD research regarding amyloid plaques have been fraught with disappointment. Several years focusing on amyloid plaques, the hallmark of AD ultimately resulted in no significant progress toward therapeutic strategies and prevention^13^. Recently, pharmaceutical companies have announced that their trials of drugs meant to help block the production of amyloid plaques had failed as AD patients receiving the drugs got worse instead^14^. The outcome is considered to be disappointing. Thus, though research has shown time and time again that amyloid plaques may have a crucial role in AD, it is not the only key factor. Some researchers have turned their focus to neuroinflammation and believed that it may be the big breakthrough as the main target because it is killing the bulk of the neurons that leads to AD^15^. However, neuroinflammation has a role in AD is not a novel concept, researchers have been studying its role for a while. In 2013, researchers published a research that examined postmortem brains^11^. All of the postmortem brains had evidence of tau proteins and amyloid plaques, both are hallmarks of AD. But only ~50% of the individuals had pathological cognitive decline when they were alive. The other half had normal cognitive function. The only difference found was the inflammatory response. There were more inflammatory cells in the brain in the individuals who had pathological cognitive decline versus those who had normal cognitive function, further suggesting that inflammation has a crucial role in dementia^16,17^.

Several studies showed that HLA genes are associated with AD^18,19^. Smith et al. demonstrated that β2-microglobulin, a component of MHC I molecules, could negatively regulate cognitive function in the hippocampus with an age-dependent pattern. Furthermore, systemic β2-microglobulin accumulating in aging blood may promote age-associated cognitive function decline and impair neurogenesis through MHC I^6^. Wang et al. identified HLA gene variants including TNF-α rs2534672, rs2395488, HFE rs1800562, and RAGE rs2070600 might involve in the structural change of brain regions that are associated with AD, thus modulating the susceptibility of AD^7^. In this study, we identified the HLA genotypes of AD patients and compared with general population within the same ethnic groups. Although HLA class I genotype distribution is significantly different between AD and general population, HLA-A*11:01, HLA-B*40:01, and HLA-C*07:02 are the most major genotypes for both groups. We also identified that HLA-A*02:01 was associated with the age of AD onset, but as HLA genotypes are too diverse and the sample size is small, this finding is only for reference and HLA genotypes could not be used as a practical biomarker. The major contribution of this study is that we found that self-antigen load was significantly associated with age of AD onset. This result is compatible with the hypothesis that germline mutation generated self-antigens did have a negative role in AD, as early-onset patients have significantly higher self-antigen load.

Auto-antibodies could be produced under both physiological and pathophysiological situations. Under normal physiological situation, humans will generate natural auto-antibodies to recognize self-antigens in order to promote the identification and clearance of dead and dying cells^20^. However, under pathophysiological situation, high affinity auto-antibodies will be produced. The mechanism of high affinity auto-antibodies production is mainly through inflammatory responses that activate affinity maturation of antibody through targeting self-antigens^21^. Through binding to high affinity self-antigens, pathogenic auto-antibodies launch and retain the immuno-inflammatory cascade responsible for tissue damages. Though the CNS is previously considered to be an immune privileged site, recent research has shown that a defined lymphatic and glympathic system is capable of draining self-antigens and initiate CNS-directed adaptive immunological reactions^15^. The trigger of T-cell mediated adaptive immunological reactions against self-antigens depends mainly on antigens drainage to structures like the lymph nodes of deep cervical chain, the leptomeningeal compartments and the choroid plexus^22^. Therefore, T cells are capable of infiltrating the CNS and influence immune homeostasis in a pathogenic manner^23^. When immunological disturbance is presented in the CNS, T cells may become pathogenic when inflammation persisted^24^. As to the age of AD onset, only 4–5% AD patients develop before age 65, the so called early-onset AD^25^. At present, the cause of early-onset AD is not clear. Mutations of several genes including amyloid protein precursor (APP), presenilin-1 (PSEN1), and presenilin-2 (PSEN2) are thought to be responsible for autosomal dominant forms of early-onset AD. All these genes are involved in generation of β-amyloid^26^. However, most early-onset AD is sporadic and only around 4–5% of patients with early-onset AD have an autosomal dominant mutation in APP, PSEN1, and PSEN2 gene. Recent research indicates that early-onset AD is quite a heterogeneous disorder^27^. Based on our finding, it is reasonable to state that besides those well known genetic mutations involved in generation of β-amyloid, more-severe neuroinflammation may also contribute to the earlier onset of AD, as the self-antigen load estimated in this study is with strong-binding affinity, which is considered to be pathogenic.

To our knowledge, this is the first study based on NGS whole-exome sequencing to study germline mutation derived self-antigen load in AD. Our finding indicated that self-antigen load did have a role in the pathogenesis of AD probably owing to its association with neuroinflammation. This finding may also contribute to further research, regarding disease mechanism and development of novel biomarkers or treatment.

## Supplementary information

Supplementary Figure Legend

Supplementary Figure 1

## Data Availability

Whole-exome sequencing data and de-identification clinical data were available on http://ad.stat.sinica.edu.tw.

## References

[1] Karch CM, Goate AM (2015). Alzheimer’s disease risk genes and mechanisms of disease pathogenesis. Biol. Psychiatry.

[2] Heneka MT (2015). Neuroinflammation in Alzheimer’s disease. Lancet Neurol..

[3] Rosenberg RN (2016). Genomics of Alzheimer disease: a review. JAMA Neurol..

[4] Raghavan N, Tosto G (2017). Genetics of Alzheimer’s disease: the importance of polygenic and epistatic components. Curr. Neurol. Neurosci. Rep..

[5] Smith LK (2015). β2-microglobulin is a systemic pro-aging factor that impairs cognitive function and neurogenesis. Nat. Med..

[6] Wang ZX (2017). Genetic association of HLA gene variants with MRI brain structure in Alzheimer’s disease. Mol. Neurobiol..

[7] Steele NZ (2017). Fine-mapping of the human leukocyte antigen locus as a risk factor for Alzheimer disease: a case-control study. PLoS Med..

[8] Lu RC (2017). Association of HLA-DRB1 polymorphism with Alzheimer’s disease: a replication and meta-analysis. Oncotarget.

[9] Padmadas N, Panda PK, Durairaj S (2018). Binding patterns associated Aß-HSP60 p458 conjugate to HLA-DR-DRB allele of human in Alzheimer’s disease: an in silico approach. Interdiscip. Sci..

[10] Schetters STT, Gomez-Nicola D, Garcia-Vallejo JJ, Van Kooyk Y (2018). Neuroinflammation: microglia and T cells get ready to tango. Front Immunol..

[11] Perez-Nievas BG (2013). Dissecting phenotypic traits linked to human resilience to Alzheimer’s pathology. Brain.

[12] Huang P (2019). REST rs3796529 genotype and rate of functional deterioration in Alzheimer’s disease. Aging Dis..

[13] Selkoe DJ (2019). Alzheimer disease and aducanumab: adjusting our approach. Nat. Rev. Neurol..

[14] Panza F (2019). Are antibodies directed against amyloid-β (Aβ) oligomers the last call for the Aβ hypothesis of Alzheimer’s disease?. Immunotherapy.

[15] Louveau A (2015). Structural and functional features of central nervous system lymphatic vessels. Nature.

[16] Weisová P (2019). Therapeutic antibody targeting microtubule-binding domain prevents neuronal internalization of extracellular tau via masking neuron surface proteoglycans. Acta Neuropathol. Commun..

[17] Mandler M (2019). Effects of single and combined immunotherapy approach targeting amyloid β protein and α-synuclein in a dementia with Lewy bodies-like model. Alzheimers Dement..

[18] Heneka MT, O’Banion MK, Terwel D, Kummer MP (2010). Neuroinflammatory processes in Alzheimer’s disease. J. Neural Transm..

[19] Heneka MT, O’Banion MK (2007). Inflammatory processes in Alzheimer’s disease. J. Neuroimmunol..

[20] Wu J, Li L (2016). Auto-antibodies in Alzheimer’s disease: potential biomarkers, pathogenic roles, and therapeutic implications. J. Biomed. Res.

[21] Chitnis T, Weiner HL (2017). CNS inflammation and neurodegeneration. J. Clin. Invest.

[22] Suzuki K, Iwata A, Iwatsubo T (2017). The past, present, and future of disease-modifying therapies for Alzheimer’s disease. Proc. Jpn .Acad. Ser. B Phys. Biol. Sci..

[23] Herline K, Drummond E, Wisniewski T (2018). Recent advancements toward therapeutic vaccines against Alzheimer’s disease. Expert Rev. Vaccines.

[24] Di Benedetto G (2019). Beneficial effects of curtailing immune susceptibility in an Alzheimer’s disease model. J. Neuroinflamm..

[25] Mendez MF (2019). Early-onset Alzheimer disease and its variants. Continuum.

[26] Gao Y (2019). Mutation profile of APP, PSEN1, and PSEN2 in Chinese familial Alzheimer’s disease. Neurobiol. Aging.

[27] Wingo TS (2019). Association of early-onset Alzheimer disease with elevated low-density lipoprotein cholesterol levels and rare genetic coding variants of APOB. JAMA Neurol..

