# Supplementary material for: Association of early-onset Alzheimer’s disease with germline-generated high affinity self-antigen load
Source: Transl Psychiatry. 2020 May 12;10:146. doi: 10.1038/s41398-020-0826-6 (PMC7217838; doi:10.1038/s41398-020-0826-6)
Supplement: Supplementary file 1 — Supplementary Figure Legend [file 41398_2020_826_MOESM1_ESM.docx]

Supplementary Figure 1.

ROC curve analysis for the cut-off value estimation of the high self-antigen load.
