# Supplementary figures and images for: Association of early-onset Alzheimer’s disease with germline-generated high affinity self-antigen load
Source: Transl Psychiatry. 2020 May 12;10:146. doi: 10.1038/s41398-020-0826-6 (PMC7217838; doi:10.1038/s41398-020-0826-6)

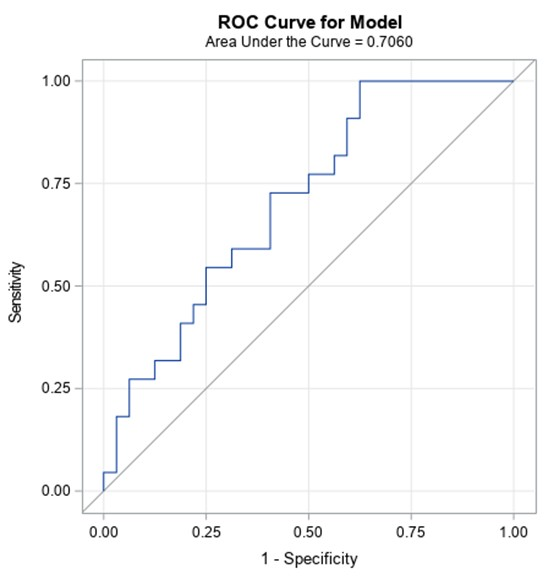

Supplement: Supplementary file 2 — Supplementary Figure 1 [file 41398_2020_826_MOESM2_ESM.tif]
